# Victims of Infanticide and Conspecific Bite Wounding in a Female-Dominant Primate: A Long-Term Study

**DOI:** 10.1371/journal.pone.0082830

**Published:** 2013-12-18

**Authors:** Marie J. E. Charpentier, Christine M. Drea

**Affiliations:** 1 Department of Evolutionary Anthropology, Duke University, Durham, North Carolina, United States of America; 2 Department of Biology, Duke University, Durham, North Carolina, United States of America; 3 CEFE-CNRS, UMR 5175, Montpellier, France; University of Tasmania, Australia

## Abstract

The aggression animals receive from conspecifics varies between individuals across their lifetime. As poignantly evidenced by infanticide, for example, aggression can have dramatic fitness consequences. Nevertheless, we understand little about the sources of variation in received aggression, particularly in females. Using a female-dominant species renowned for aggressivity in both sexes, we tested for potential social, demographic, and genetic patterns in the frequency with which animals were wounded by conspecifics. Our study included 243 captive, ring-tailed lemurs (*Lemur catta*), followed from infancy to adulthood over a 35-year time span. We extracted injury, social, and life-history information from colony records and calculated neutral heterozygosity for a subset of animals, as an estimate of genetic diversity. Focusing on victims rather than aggressors, we used General Linear Models to explain bite-wound patterns at different life stages. In infancy, maternal age best predicted wounds received, as infants born to young mothers were the most frequent infanticide victims. In adulthood, sex best predicted wounds received, as males were three times more likely than females to be seriously injured. No relation emerged between wounds received and the other variables studied. Beyond the generally expected costs of adult male intrasexual aggression, we suggest possible additive costs associated with female-dominant societies – those suffered by young mothers engaged in aggressive disputes and those suffered by adult males aggressively targeted by *both* sexes. We propose that infanticide in lemurs may be a costly by-product of aggressively mediated, female social dominance. Accordingly, the benefits of female behavioral ‘masculinization’ accrued to females through priority of access to resources, may be partially offset by early costs in reproductive success. Understanding the factors that influence lifetime patterns of conspecific wounding is critical to evaluating the fitness costs associated with social living; however, these costs may vary substantially between societies.

## Introduction

Behavior is the phenotype by which animals interact with their physical and social surroundings and, like any phenotype, is shaped by natural selection. Consequently, variation in social behavior may profoundly impact individual fitness. For instance, affiliative relationships may confer fitness benefits through their impact on infant survival [1] or on adult coalitionary support in within-group contests [2]. The aggression an animal receives from conspecifics constitutes an obvious form of social behavior that is likely to vary significantly between members of different societies and between individuals across their lifetime, with potentially dramatic consequences on individual fitness. Indeed, aggression received in infancy is widespread and often fatal [3], [4], [5]. Likewise, in a variety of taxa, escalated fights between conspecific adults can result in death [6]. Even non-lethal injuries inflicted by conspecifics may involve energetic expenditure [7], constraints on feeding efficiency [8], reduction in competitive ability [9], missed mating opportunities [10], and increased predation risk [11]. Although we have identified many triggers of aggression, as well as their associated costs, we still understand little about the sources of variation in received aggression that underlie lifetime patterns in conspecific wounding. Notably, the relevant long-term data are difficult to obtain and ephemeral aggressive encounters often escape detection. Here, we used the unique, long-term, life-history data and health records from a captive colony of ring-tailed lemurs (*Lemur catta*) – a female-dominant, strepsirrhine primate – to extract reliable frequencies of conspecific bite wounding as a proxy for received aggression. We then examined the social, demographic, and genetic correlates of those animals receiving bite wounds during both infancy and adulthood.

The study of aggression in humans and other animals has had a long history [12], [13], particularly focused on explaining the showy combats or excessive violence of males [14], [15], [16]. Although overt aggression is almost always greater in males than in females [17], female aggression is not negligible, often occurring in the context of competition over resources or reproductive rights [18], [19], [20], [21]. Although strangers evoke particularly strong aggressive responses, much aggression also occurs between familiar individuals (reviewed in: [22]). It is within this latter context of individualized societies that we explore variation in aggression received.

Notably, in many primate societies characterized by long-term bonds and dominance hierarchies, an animal's demographic traits and social standing are likely to predict its aggressive interactions. For instance, in typical male-dominant species, the highest-ranking males are expected to receive the brunt of aggressive challenges [23], especially in competition over access to reproductive females [24]. As a consequence, adult males are sometimes known to assume the greatest costs of their high-risk behavior [25]. We appreciate far less, in these societies, about variation in female aggression and female intrasexual competition (reviewed in: [26]) or even about the main targets of female aggression. In humans, for example, indirect aggression (such as social exclusion or ostracism) is greater in adolescent girls compared to boys [13] and, although mild, women generally direct their aggression against other females, often when competing for men [27]. In primates, more generally, female aggression is best understood in terms of species differences in inter- and intra-group resource competition (e.g. [2], [28]), but less so in terms of individual variation within a species.

To address this gap in our understanding, we focus on one species in which both sexes are ‘at risk’ of receiving significant aggression. Notably, if being at the top of the hierarchy predisposes an animal to receive aggressive challenges or assume the greatest risks, then one might expect that, compared to females in male-dominant societies, females in female-dominant societies suffer relatively greater costs associated with aggression. For instance, in the spotted hyena (*Crocuta crocuta*) – a carnivoran that portrays a primate-like, but female-dominant, social organization [29]– females are more likely than males to lead clan wars [30]. We therefore selected as our study subject a species known both for its male aggression and for its unusually ‘masculinized’ female behavior [31].

**Figure 1:**
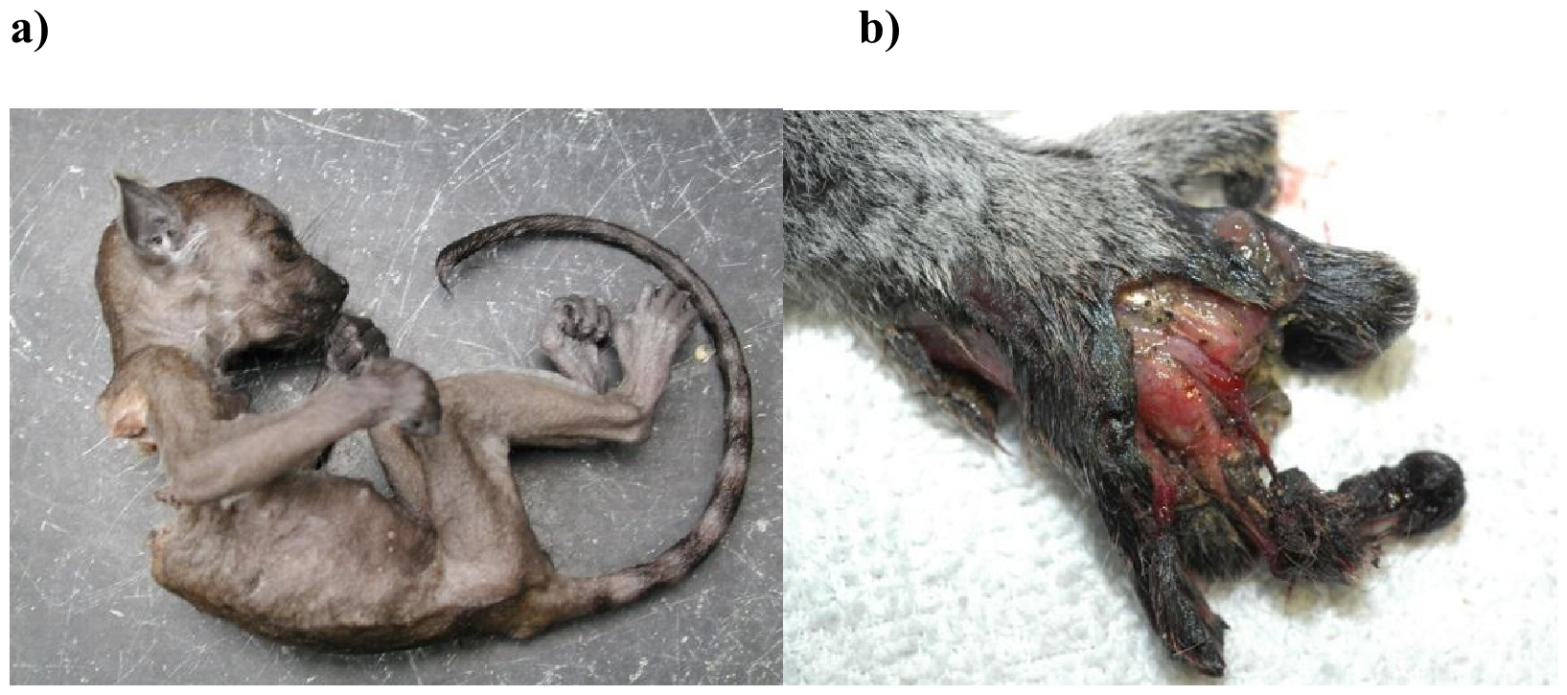
Conspecific bite wounds in ring-tailed lemurs. (a) Bite wounds received in infancy are typically lethal, involving more force than necessary to kill the infant. The attack producing this infanticide occurred coincident with a rank reversal between two sisters, the older of which was the dam of the deceased infant; the younger of which was the suspected attacker. (b) Hand injuries of an adult male, inflicted by another adult male in an adjacent enclosure. These males had shared the same forest enclosure for several months, but were physically separated at the onset of the breeding season to prevent aggression. These injuries were incurred, through the fence, post separation, (Photos by C. Drea, courtesy of the Duke Lemur Center).

Ring-tailed lemurs occur in multi-male multi-female groups, characterized by female social dominance over males, female philopatry, and a strictly seasonal, but promiscuous breeding system [32]. They show significant intra- and intersexual aggression, both in captivity [31], [33] and in the wild [32], [34], [35], [36]. Likewise, confirmed sightings of infanticidal attacks have been reported for this species under both captive and field conditions [33], [37], [38], [39]. Because fights in any species are often brief, whereas wounds remain visible for at least several days, researchers or caretakers are more likely to observe and record the injurious consequences of aggression than the aggressive event itself. We therefore considered bite wounds to be a good proxy of the serious aggression an animal receives over its lifetime. Although more subtle harassment could also impact individual fitness, bite wounds represent the most extreme subset of aggression that is most likely to be selective (see Figure 1). Accordingly, we investigated the potential influence of multiple variables on the frequency of bite wounds received by captive, semi-free-ranging animals at different stages of their life.

Reports of infanticide in lemurs have been focused on the aggressors, as is typically the case, with adaptive explanations including the elimination of resource competitors for females or sexual selection on males [37]. More unusually, however, beyond implicating extra-troop males, infanticide in lemurs has implicated both male and female troop members, including relatives [37], [38], [39]. Because infanticidal attacks can be performed by all categories of adults, we directed our focus, here, to the characteristics of the victims. We reasoned that a mother's experience of her ecological and social environment could greatly influence her offspring's development and fitness [40], including via her protection against infanticide. From this ‘victim’ perspective, we expected that either maternal experience (as revealed by parity) or overall social experience (as revealed by age) would be the main predictor of infant survival. We also expected that mothers of twins might be less efficient than mothers of singletons at defending their offspring against injurious attacks. Lastly, we considered the sex of the offspring and offspring's genetic diversity (heterozygosity, measured with microsatellite markers, see for example: [41]) as potential additional sources of variance. We included heterozygosity because researchers working with a variety of taxa have documented effects of genetic variation on social behavior, including a relationship between heterozygosity and aggression [42], [43], [44], [45].

In adult lemurs, aggression also can be serious, even deadly, in either sex. Inter-group aggression among females is extremely high [34], [36], [46], [47], [48], with resident females often taking the lead in group defense [32]. Likewise, in intra-group aggression, dominant females are responsible for the greatest number of aggressive events [49] and are often observed to aggressively target and harass subordinate females [37], [50], [51], ultimately evicting them from the group [52], [53]. Male-male aggression is especially pronounced during the reproductive season, both in the wild and in captivity [31], [32], [35], when males additionally receive aggression from non-receptive females [36]. Females also often target males attempting to immigrate during the birth season [33], and even have been known to kill adult males (unpublished observations).

As with our analyses of wounding in infancy, we focused our analyses of wounding in adulthood on the characteristics of the injured animals, rather than on their attackers. We expected that immigrant (i.e., nonnatal) individuals, who may have been less well socially integrated within the group, would suffer higher wounding pressure. Moreover, given the aggressivity of females, we did not necessarily predict the same degree of male-biased sex differences in bite wounds as would normally occur in male-dominant species (e.g. [54]). Although lemurs are sexually size monomorphic and our study population showed relatively little weight variation in adulthood [55], we nonetheless examined the relationship between weight and the occurrence of bite wounds in adults. Lastly, we examined the potential effects of social housing conditions (e.g. group size) and individual genetic quality on bite wounds received by adult ring-tailed lemurs. Evidence of a relationship between wounds and any of these variables would shed new light on how bite wounds might variably impact individual fitness.

## Methods

### Study population and housing

Our subjects represented a total of 243 ring-tailed lemurs of all ages (including 110 females, 118 males, and 15 animals of undetermined sex), studied over a 35-year period. All of the animals were members of a captive colony at the Duke Lemur Center (DLC) in Durham, North Carolina. Since its inception in 1966, the DLC has maintained a full-time animal caretaker and veterinary staff. Most of the subjects have been housed socially, in large forested enclosures (3–7 ha), which were often connected to allow males to transfer between groups [33]. Thus, most of the animals are semi-free ranging and can forage freely, but have access to indoor, temperature-controlled areas, where they can avoid inclement weather (and see for details [31]). A minority of the animals occupies indoor/outdoor enclosures, with one or a few companions, year round. These individuals are provided with natural vegetation and natural forage, as well as various environmental enrichment items (ropes, toys, etc.). All of the subjects are additionally fed a daily ration of a commercially available primate diet (Purina® Monkey Diet 5038, PMI Nutrition International, Inc., Brentwood, MO 63144, USA), supplemented with fresh fruits and vegetables. Water is always available.

Given the social housing conditions, aggressive interactions between animals occur naturally. As the animals are individually known and monitored daily at close range, all but the most minor of injuries are noted. Specifically, if any aggressive physical contact is seen or evidence of such interaction is found (such as blood stains or tufts of hair) then the animals or most likely culprits (based on dyadic behavior, hair pulls, repeated licking, subdued or timid behavior, etc.) are caught up and examined in-hand to check for any sign of injury. Details on the extent and type of wound are recorded (see below). In general, the size of the forested enclosures allows aggressively targeted adults to readily avoid or escape their attackers [56]; nevertheless, in the case of relentless harassment or severe wounding, targeted animals are removed from their group [33].

### Life-history records and databases

Life-history and health records, specifically including information on infant wounds and all mortality, have been maintained for DLC animals since the establishment of the colony; however, animals born between 1966 and 1971 were either born elsewhere or had missing data (e.g. the mother's age was unknown). To minimize missing data, we included as subjects only those animals born at (N = 237) or acquired by (N = 6) the DLC between 1971 and 2006. Our analysis of bite wounds received in infancy, based on routine monitoring, thus spanned the full 35 years of our study (see below). For our analyses of bite wounds received later in life (which were mostly non-lethal, including bleeding lacerations and punctures), we relied on the medical records database (MedARKs, Medical Record Keeping system, software; ISIS) that was implemented at the DLC in 1994 [41] for details). Unlike records on animal death, records on non-lethal bite wounding were routinely maintained from this date forward. Thus, our analysis of bite wounds received in adulthood spanned the last 12 years of our study period (from 1994 to 2006).

Our analysis of bite wounds received in infancy (0–1 yr) involved the majority of our subjects, namely the pool of 237 lemurs (including 109 females, 113 males, and 15 animals of unknown sex), born at the DLC from known mothers that either survived their first year (N = 173), received (and inevitably died from) bite wounds (N = 15) or died before one year of age from unknown or unconfirmed reasons (e.g. suspected predation) other than conspecific bite wounding (N = 49). For the age span approximating juvenility (1–2 yrs), there was only one record of a non-lethal bite wound. Owing to the small sample size, we excluded this age category from our analyses. Our analysis of bite wounds received in adulthood involved 45 lemurs (including 20 females and 25 males; aged 2.4–20.1 yrs). The availability of records on wounding events, as opposed to observed aggressive interactions, precluded a long-term analysis of the aggressors' characteristics. To address the latter, we relied on a 2-year behavioral study, conducted toward the end of the period covered by the current study, that was focused specifically on observed aggression occurring within a subset of our study population [31].

### Ethics statement

As this study covers a 35-yr time span, animal housing and handling practices varied somewhat throughout the study period (see above); however, all research protocols were approved by Duke University's IACUC (currently, DLC protocol #A203-11-08 covers behavioral observations and DLC protocol # A027-12-02 covers all procedures that DLC staff perform to assist researchers). Our own protocols at the time of the study (relevant to cover the procurement of banked blood and health records) were also approved by Duke University's IACUC (protocols #A245-03-07 and #A232-06-07). The DLC is fully accredited by the American Association for the Accreditation of Laboratory Animal Care. Animal care met with institutional guidelines and was in accordance with USDA regulations. Additional information about the DLC, including its education, conservation, and research mission, can be found at http://lemur.duke.edu/.

### Genetic analyses

We genotyped 76 (32%) of the 237 infants and 45 adults studied. Because the pedigree of the lemur colony was unresolved, we considered the mean neutral heterozygosity per individual (Ho) as a proxy of inbreeding or genetic quality. We used 7–14 microsatellite loci per individual (mean genotyped loci per individual ±s.e.m.: 13.54±0.14; see: [41] for details). Ho was calculated as the number of heterozygous loci divided by the number of genotyped loci, and ranged from 0.21 to 0.86 (mean ±s.e.m.: 0.56±0.01). Mean Ho in our study population is lower than that found in wild populations of *L. catta* (e.g., [57]). Nevertheless, because the DLC colony has comprised both relatively inbred and relatively outbred individuals, Ho was previously shown to be a good estimate of genome-wide inbreeding for this population [41], [58]. Moreover, we re-ran our analyses using two other estimates of heterozygosity, including standardized heterozygosity (SH: [59] and internal relatedness (IR: [60]; our results did not change with these estimates (data not shown).

### Co-variables and statistical analyses

#### Correlates of bite wounds in infants

Using a first subset of the infant population (comprising N = 173 live infants and N = 15 infants that died from bite wounds, but excluding 49 individuals that died of other causes before one year of age), we examined the effects on early mortality due to conspecific bite wounds of the following five variables: First, we considered the mother's parity (primiparous *vs*. multiparous; for which three dams had unknown status) and, second, the mother's age at her infant's birth. Age at first conception in our females, as in other captive studies [31], [61], occurred somewhat earlier than in the wild [62], and ranged from 1.72 to 8.20 years (mean ±sem: 2.71+0.21). Third, we considered the litter size (twin *vs*. singleton) and, fourth, the infant's sex (male *vs*. female, with one infant of unknown status). Lastly, despite our limited sample size of genotyped infants that died from bite wounds (N = 4 over 15 deaths), we also examined the effect of the infant's Ho on its probability of being lethally attacked.

Using the same covariables as above, we then performed two additional analyses, using different subsets of the infant population. First, we included all infants that died before one year of age (N = 64; total sample size  = 237) and, second, we included infants that died before one year for any reason other than bite wounds (N = 49; total sample size  = 222). We used Generalized Linear Models (Proc GENMOD, SAS version 9) with a binomial distribution (with logit-link function) because our measure of mortality included values of either 0 (for any individual that survived beyond one year of age) or 1 (for any individual that died before one year of age). Because most mothers contributed more than one offspring to the dataset, we used repeated measures on the mother's identity to handle the potential issue of non-independence of data points. Because we had missing information for some covariates, especially for the infant's Ho, we used a backward model selection procedure to select a best-fit set of explanatory variables. Specifically, we started with all potential explanatory effects incorporated into the model and then removed first the infant's Ho. By doing so, our sample size almost tripled, as we had so few genotyped infants. Then, we removed the effect with the highest *p* value from the model. We repeated these steps until all *p* values for individual parameters remaining in the model were less than 0.05. Using the Quasi-likelihood under the Independence model Criterion (QIC, an analogous of the AIC, designed for models fit by generalized estimating equations; [63]), we further confirmed that all the intermediary models showed a better fit (smaller QIC) to the data than had the previous ones, resulting in the selection of the best final model.

Finally, to ensure that none of our covariates were too correlated to be considered altogether in the same model (e.g., parity and mother's age are mildly correlated: R^2^ = 21%), we tested for multicolinearities in our data set (Proc REG SAS version 9). The variances of inflation we obtained were inferior to 1.4 for all considered variables, and are values inferior to the threshold of 10, as required; all four indices of condition varied between 1.7 and 6.1, and are also values inferior to 10, as required; lastly, the eigenvalues of all four variables were between 0.09 and 1, which are also acceptable values (>0.01) [64].

#### Correlates of bite wounds in adults

We examined the effect of five variables on the total number of bite wounds received over an adult's lifetime or during the study period. First, as a measure of the effect of social integration, we looked at whether the victim was a natal member of its social group or an immigrant, nonnatal member. These immigrant individuals were not born at the DLC, but lived there possibly for many years. Second, we considered the sex of the wounded party. Our third variable was the mean ‘weight-for-age’ of each animal during the study period. Because each individual had multiple weight measurements taken during the study period, we considered the mean of the residuals of weight, calculated following published methods ([65] see [41] for additional details). Because a given subject may have experienced different social housing conditions throughout the period of study, our fourth variable was the wounded animal's main housing experience, defined as follows: (1) most of the animal's lifespan spent as part of a semi-free-ranging, social group, (2) most of the time spent in a small enclosure with few or even no conspecifics (as even animals housed alone could be wounded, through a fence, by neighboring animals, Figure 1b), and (3) approximately equal time spent semi free-ranging as spent in small enclosures. Lastly, we studied the effect of the adult's Ho on the total number of bite wounds received. Because we pooled several years of data in our analyses, we could not consider dominance status as an additional variable. In *L. catta*, unlike in many cercopithecine primates, rank is not necessarily stable in either sex nor is it always linear (e.g. [66], [67], [68] and references within). Variability in dominance relationships within a season or across the lifespan, coupled with occasional circularity, precluded assigning representative ranks.

We used Generalized Linear Models to study the effects of our five independent variables on the number of wounds received (Proc GENMOD, SAS version 9). Because the latter was a count variable showing a variance greater than the mean, we used a negative binomial distribution with a log-link function. We confirmed the fit to this distribution using XLSTAT. Moreover, we included the total duration of data recording, which varied between individuals, as an offset in the regression (mean duration ±sem: 6.44 yrs±0.68). As in the previous analysis, we used a backward model selection procedure and confirmed that the final model showed a better fit (smaller QIC) to the data than had the previous, intermediary, model.

We conducted two final analyses based on aggression observed within a subset of these adult lemurs (N = 16). Notably, using a Spearman rank correlation test, we studied the relationship between the rate of bite wounding received during the 12-yr period covered by the present study and the rate of mild aggression received (chase, cuff, supplant, lunge, withdraw) during a separate, but concurrent, 2-yr behavioral study [31]. Our aim for this analysis was to explore the link between mild and severe aggression an animal received. Lastly, using the same 2-year behavioral data set, in which the identity of the aggressor was known, we specifically examined the biting rate of female aggressors depending on the sex of the recipient. Our aim for this analysis was to test whether a portion of the wounds received by animals might owe to female aggression, specifically, as might be predicted for a female-dominant society.

## Results

### Correlates of bite wounds resulting in infanticide

At the DLC, infant mortality within the first year, for any reason, was 27%. This percentage is at the low end of the range (30–51%) reported for wild populations [62], [69], [70], [71], [72]. Importantly, all instances of infant mortality attributable to infanticide, specifically, were confirmed cases (minimally 23.4% of all infant deaths). In infant lemurs (less than one year of age), all recorded bite wounds were lethal (Figure 1a).

We found that the mother's age at the birth of her infant significantly influenced whether or not her infant received and died from conspecific bite wounds before it attained one year of age (Table 1): Infants of younger mothers had a greater probability of dying from bite wounds than did infants of older mothers. Conversely, we found no effect of the mother's parity on conspecific bite wounds received by her infants (Table 1), even when parity was considered as the only predictor of the analysis (data not shown).

**Table 1:** Relationship between several explanatory variables and the occurrence of infanticidal bite wounds or the total number of adult bite wounds in ring-tailed lemurs.

	Explanatory variable	Sample size^1^	df	?^2^	Estimate	Standard error	P-value
	**Maternal age at infant's birth**	188	**1**	**4.21**	**0.14**	**0.08**	**0.04**
**Infanticide**	Maternal parity at infant's birth	184	1	0.94	0.69^2^	0.66^2^	0.33
	Twin birth	184	1	0.26	−0.31^2^	0.59^2^	0.61
	Infant's sex	187	1	1.76	0.65^2^	0.64^2^	0.23
	Infant's Ho (preliminary analysis)	66	1	0.32	−3.19	2.71	0.32
	Natal vs. non natal group members	45	1	2.31	−0.67^2^	0.42^2^	0.13
**Bite wounds in**	**Sex**	45	**1**	**9.10**	**0.85** 2	**0.27** 2	**0.003**
**adults**	Mean individual's weight (residual)	45	1	0.40	−0.34	0.55	0.53
	Housing condition	45	2	2.41	23.38^2^	0.27^2^	0.30

Variables included in the best-fit models are shown in bold.

^1^ Sample sizes varied because of missing data and the use of a backward selection procedure.

^2^ Value provided for the first modality of this class variable.

Although twice as many of the infants killed were male versus female, we found no significant effect of the offspring's sex, no effect of litter size, and no effect of the infant's Ho on the occurrence of bite wounds early in life (Table 1). The latter result likely reflects a lack of power, owing to a limited sample size of genotyped infants that died from bite wounding.

**Figure 2:**
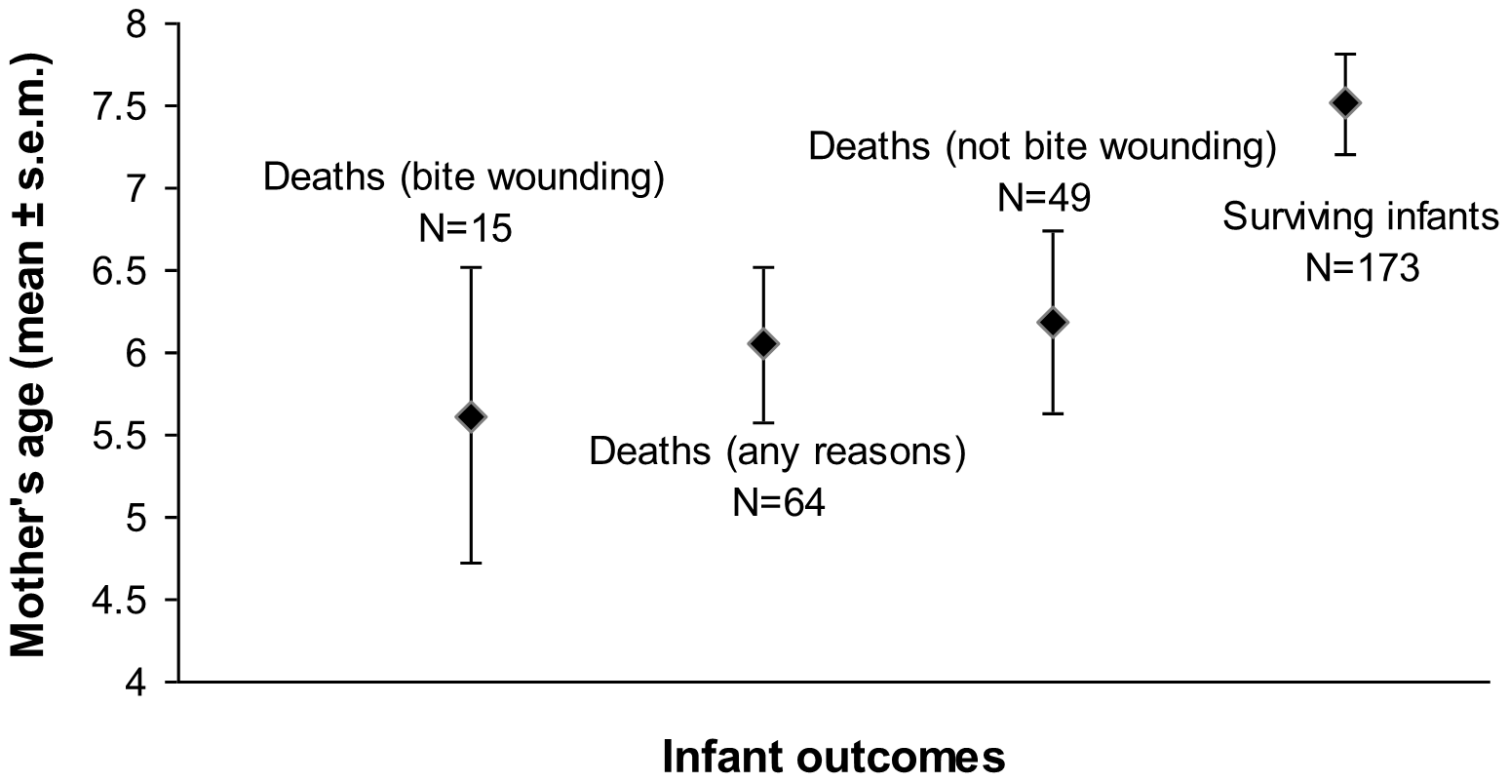
Relationship between maternal age and infant outcome (mean ±s.e.m.).

To test if the effect of the mother's youth was specific to the occurrence of infanticide, we further explored the relationship between maternal age and other infant outcomes, such as illness, accidents, or predation. We found similar results for the influence of maternal age when we considered all infants that died before one year of age, for any reason (N = 237, χ^2^ = 5.10, P = 0.024), but less so when we considered infants that died for reasons other than bite wounds (N = 222, χ^2^ = 2.79, P = 0.095): In general, infants of younger mothers suffered a greater probability of mortality than did infants of older mothers. Nonetheless, when we compared the different mean maternal ages corresponding to the different infant outcomes considered, we found the strongest age effect for the analysis of bite wounds, with mothers of infants that died from infanticide being around two years younger than mothers whose offspring survived (Figure 2).

### Correlates of bite wounds in adult lemurs

Over a 12-year period at the DLC, a total of 129 bite wounds were recorded in adults, involving 45 lemurs. Bite wounds in adults generally were not lethal, but could still be severe and would have certainly entailed fitness costs, or even the loss of life, if left untreated (Figure 1b). Indeed, one adult male was killed due to bite wounds, further illustrating the potential severity of these interactions.

**Figure 3:**
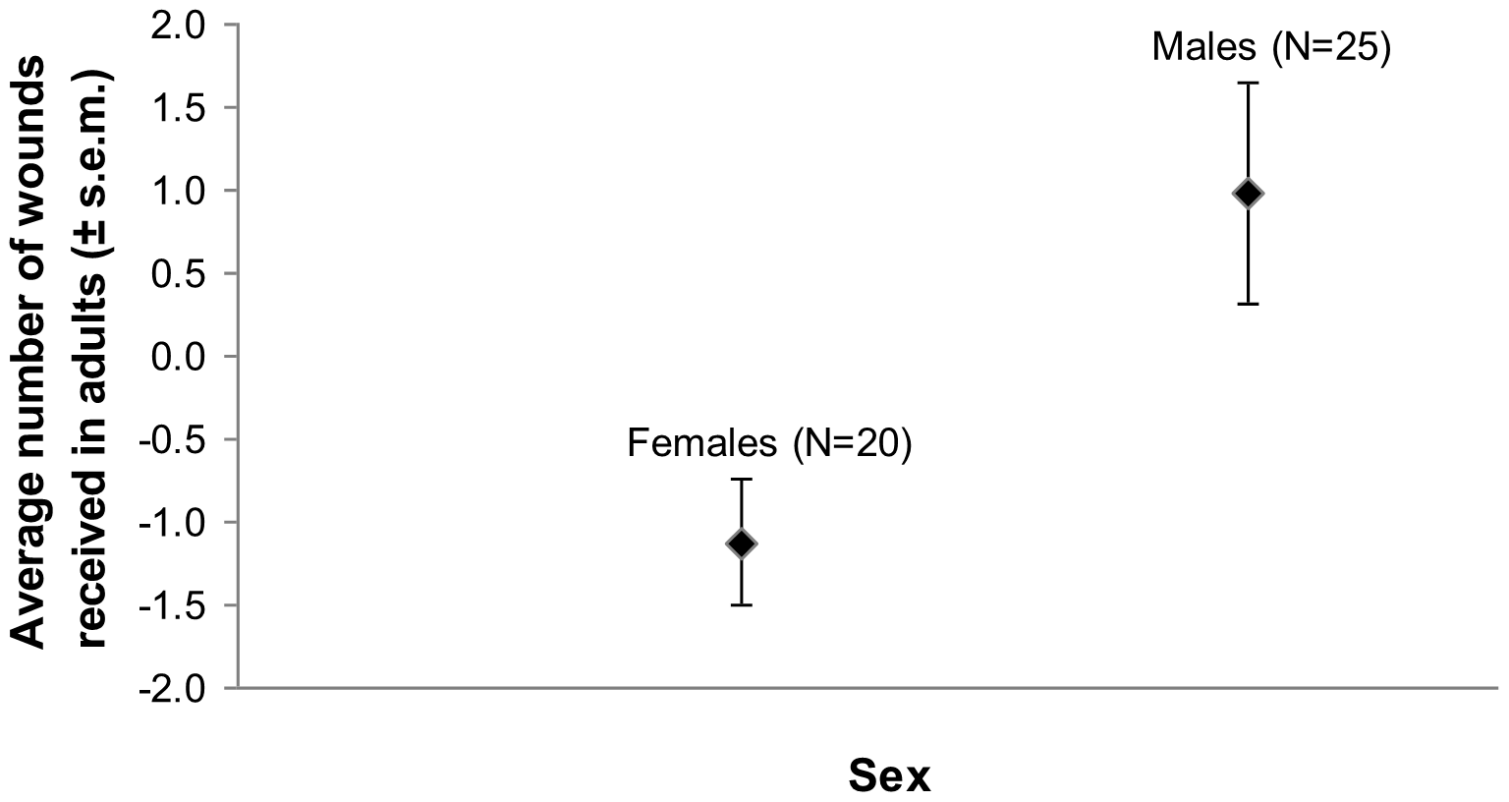
Effect of sex on the total number of bite wounds in adult ring-tailed lemurs (mean of residuals ±s.e.m.).

We detected no significant effect of social integration (natal *vs*. nonnatal group members) on bite wounds (Table 1). Likewise, we found no effects of weight, housing condition, or genetic diversity on the rates at which adult animals were wounded by conspecifics (Table 1). By contrast, we found that the total number of conspecific bite wounds an adult lemur received during its lifetime or during the period of study was influenced by its sex (Table 1): Adult males received significantly more bite wounds than did adult females (Figure 3). Although some minor bite wounds may have been missed, there is no reason to suspect that any unobserved events would have been biased in any consistent manner.

In a subset of 16 adults whose aggressive behavior was observed [31], we found a positive, but not statistically reliable, correlation between mild aggression received over a 2-year period and bite wounds received over the present retrospective 12-year period (Spearman rank correlation: rs = 048, P = 0.08). Lastly, in this same subset of animals, biting rates from female actors occurred at a rate of 0.005 bouts per hour when directed towards other females and at a rate of 0.007 bouts per hour when directed towards males.

## Discussion

In a long-lived, socially integrated species – the ring-tailed lemur – we found that different demographic parameters influenced the likelihood of receiving conspecific bite wounds in infancy versus in adulthood. In particular, lethal bite wounds received in infancy were best predicted by the age of the recipient's mother, whereas bite wounds received in adulthood were best predicted by the recipient's sex. In adulthood, both mild and severe aggression tended to be correlated across a two-year concurrent behavioral and a 12-year retrospective injury study, suggesting that bite wounds may well reflect the overall aggression an animal receives (and vice versa). Moreover, it is clear that females are responsible for inflicting a portion of these wounds. These data reveal that, in an exceptionally aggressive, female-dominant primate, both sexes incur significant costs from conspecific aggression. Notably, females suffer an initial, but substantial, fitness cost as young mothers, from the death of their infants, whereas adult males likely suffer fitness costs, over the long term, from the accumulated consequences of their wounds. The sex difference in aggression received in adulthood is consistent with field observations in this species [73], [74] and with the sex difference in adult mortality reported across various long-term, primate studies (reviewed in [25]): Presumably, greater mortality of adult males relative to adult females owes, in part, to sex differences in received aggression. By contrast, the maternal effect on aggression received in infancy is a novel and unusual finding. To our knowledge, our 35-year study represents the most complete and long-term record on the victims of infanticide in any species.

Based on this unique dataset, we document an initial cost born by young mothers that does not conform to predictions about the occurrence of infanticide in an evolutionary framework. Several adaptive advantages have been proposed for infanticide, including the exploitation of infants as a resource, parental manipulation in favor of other offspring, sexual selection between rival males [3], [75], and elimination of a competitor for resources. These purported advantages, however, do not seem to be well supported for ring-tailed lemurs, either by prior data or by the patterns we presently observed in victims. Our data are most clearly inconsistent with the first two hypotheses. In particular, although rare instances of flesh consumption have been observed in *Eulemur* [37], [76], wild ring-tailed lemurs do not appear to consume the victims of infanticide. Likewise, none of the victims of infanticide in our study had been consumed. Moreover, parental manipulation of offspring in favor of older siblings would predict an effect of parity on infanticide. In this case, the infants of multiparous females should have received more aggression than those of primiparous females, which was not supported by our current findings on maternal effects.

Although our data on the victims of infanticide cannot shed light on the perpetrators and, thus, do not relate to the purported male reproductive advantage in ring-tailed lemurs [33], [38], [39], the reproductive biology of this species does not support this hypothesis. Notably, reproduction in ring-tailed lemurs is strictly seasonal, infants are weaned prior to the onset of the next year's breeding season, and females typically breed in consecutive years [37]. Consequently, infanticide cannot influence a female's subsequent breeding potential. As previously noted [36], [77], infanticide is therefore unlikely to function as a male reproductive tactic in this species.

Lastly, although it is difficult to falsify that infanticide eliminates resource competition, according to this hypothesis, all youngsters would become potential rivals, predicting the elimination of any infant, not just the youngest infants and not just those of young mothers. Although still seemingly vulnerable, only one juvenile was killed in the 35-year period covered by this study. It may be the case that young mothers are less well equipped (socially or physically) to protect their offspring and are therefore the most often targeted. In this regard, it is interesting that many observers of infanticide report that the victim had been clinging to its mother at the time of the attack. Certainly an infant venturing out on its own should be an easier target than one that is in direct contact with its mother. As infants of multiparous females tend to spend more time off of or away from their mothers than do infants of primiparous females [78], [79], one might have expected an effect of parity on infanticide in support of this hypothesis. Specifically, one would have expected the infants of more permissive, multiparous females to receive more aggression than those of the more restrictive, primiparous females, which was not the case.

That victims of infanticide are typically observed clinging to their mothers has an alternate interpretation; namely, that the mother was the intended victim. It may be that infanticide in this species, and potentially in other lemurs, is merely a by-product, albeit a costly one, of pronounced female aggressiveness, relating both to intrasexual conflict and to social dominance over males. Because female intersexual dominance entails aggressive female interaction with extra-troop males, infanticide by males during group encounters [37] may be conservatively explained by the defensive role of female mothers in the aggressive targeting of strangers. Moreover, among strepsirrhines, inter-group encounters that involve female-female aggression are noteworthy in ring-tailed lemurs [47], [68]. Researchers have reported that such encounters increase substantially during the birth season and vary in intensity, from simple staring, to serious wounding of adult females, to even the loss of their infants. With regard to intrasexual female aggression, attacks on infants by female group members also occur in the context of attacks on their mothers, in various species (e.g. [76]), and seem to have a rank-related component, with more attacks of subordinate mothers by dominant female group members [37]. Although we cannot comment on rank-related patterns, our interpretation is consistent with observations, both from the field and from our own study population, that infants are killed in the context of inter-group or intra-group aggression involving their mothers.

Infanticide in the context of other aggression may be seen as a by-product bearing no adaptive explanation, as discussed in Jolly et al. 2000 (p. 36). We provide new data to show that the reproductive costs of infanticide are not uniformly born by all mothers, as might otherwise be predicted in support of adaptive hypotheses. Instead, these costs are greatest in young, vulnerable females. Indeed, the overall social experience of the mother (i.e., youth), rather than maternal experience more specifically (i.e., parity), was our only correlate of infant death. Young animals are often risk takers [80], [81], involved in efforts to climb the social ladder [82], [83], [84], and hence, may be more frequent targets of aggression. Moreover, they may be less experienced in defending themselves or their infants during aggressive encounters. The combination of youth and possible aggressive proclivities could prove lethal to their infants.

The costs of female dominance may also be observed in adulthood. Perhaps females of female-dominant species are more at risk of injury in adulthood than are females of male-dominant species, but the comparative data to adequately address this suggestion are lacking. Nonetheless, with regard to males, a significant sex difference emerged in the frequency with which these animals received aggression. Not surprisingly, in male-dominant species, males are likely to receive more aggression than are females: During conflicts, male baboons are wounded, on average, 1.4 times more frequently than are females [54]; male macaques are wounded, on average, twice as often as are their female counterparts [85]; aggression received by male mangabeys is about seven times greater than aggression received by females [86]. By comparison, we found that male ring-tailed lemurs were about three times more likely to be seriously injured than were female conspecifics. Thus, female dominance in this species did not alter the sexually dimorphic pattern of received aggression typically observed in male-dominant, primate species, but it more likely contributed to the incidence of male injuries. Our finding on bite wounds in adulthood is consistent with a prior finding that male *L. catta* receive over three times as much aggression as do females [31]. Moreover, our examination of biting rates displayed by known aggressors suggests that females are responsible for at least a portion of the wounds experienced by animals of both sexes. Intrasexual male competition to gain access to fertile females is fierce in *L. catta* [87], but could be on a par with that displayed by males of other aggressive species. Male lemurs therefore suffer the high costs of intrasexual aggression experienced by other species, but possibly coupled with the additional costs of intersexual aggression.

Our findings highlight the critical importance of socio-demographic factors in influencing received aggression among the members of individualized societies, particularly in species characterized by sex-role reversal. Aggression in females may have selective advantages, but often comes at a reproductive cost (e.g. [88], [89]). Indeed, female dominance in the spotted hyena is associated with a specific reproductive cost, namely the death of first-born infants, associated with the hormonal mechanism of anatomical masculinization [90], [91]. By comparison, in lemurs, the reproductive cost of infanticide born by young mothers may be associated with the hormonal mechanism of behavioral masculinization [66]. Beyond the increased potential for a young female's aggressive encounters to entail her infant's death, important costs of female dominance and aggressiveness are also assumed by males, notably through the female's contribution to the high rate of wounding in adult males. Therefore, intrinsic life-history characteristics of the individual affect its behavioral interactions, the consequences of which can profoundly impact individual fitness.
